# RCT evidence suggests that solids introduction before age 6 months does not adversely impact duration of breastfeeding

**DOI:** 10.1111/mcn.13029

**Published:** 2020-06-16

**Authors:** Paul J. Turner, Michael R. Perkin

**Affiliations:** ^1^ Section of Inflammation, Repair and Development, National Heart & Lung Institute Imperial College London South Kensington London SW7 2AZ UK; ^2^ Population Health Research Institute St. George's, University of London London UK

Dear Editor,

We read with interest the recent publication by Lessa et al. (2020), who conducted a secondary analysis of data from 3 UK‐based cohorts and reported that the earlier introduction of solids into the infant diet was associated with a shorter duration of breastfeeding. These data are relevant, given current discussions as to the timing of introduction of solid foods into the infant diet as a strategy to reduce the incidence of food allergy and potentially life‐threatening anaphylaxis (Turner, Feeney, Meyer, Perkin, & Fox, 2018).

The authors state that “only controlled trials can truly test whether the relationship between the age of first solids and breastfeeding is causal, and such trials are rare.” Unfortunately, the authors fail to include the largest published randomised controlled trial (RCT) of early solid food introduction, the EAT (Enquiring About Tolerance) Study, which randomised 1,303 mothers and their infants to either introduction of six allergenic foods from 3 months of age alongside continued breastfeeding, or standard UK government advice (exclusive breastfeeding until around 6 months). In the intervention group, 97.2% (593/610) of infants were still breastfed at 6 months, compared with 97.8% (619/633) in those randomised to solids introduction after age 6 months, with no impact on duration of breastfeeding by intention‐to‐treat analysis (Perkin et al., 2016). As an RCT, the EAT study avoids the issue of reverse causality associated with cohort studies and clearly demonstrates that in this cohort, solids introduction prior to age 6 months occurred without adversely affecting breastfeeding.

Lessa et al. conclude that “early introduction of solid feeding predicts a shorter breastfeeding duration and suggests that deferring solid feeding [to the age of 6 months] is important to sustain breastfeeding.” However, there are now high‐quality data from numerous RCTs that the delayed introduction of peanut and egg increases the risk of developing allergy to these foods, and the EAT study demonstrated that solids introduction does not adversely affect breastfeeding. These data resulted in the Committee on Toxicity of Chemicals in Food, Consumer Products and the Environment (an independent scientific committee providing advice to UK Government Departments) concluding that there is moderate quality evidence for the introduction of egg between 4 and 6 months of age as a strategy to reduce the risk of egg allergy (COT, 2016).

## CONFLICTS OF INTEREST

MRP and PJT have received grants from the Food Standards Agency (FSA), National Institute for Health Research (NIHR), and the Medical Research Council (MRC). PJT provided expert opinion to the FSA, Public Health England, and Departments of Health in relation to timing of introduction of solids introduction into the infant diet. MRP was the Chief Investigator for the EAT study. Both MRP and PJT contributed to national guidance from the British Society for Allergy & Clinical Immunology on the topic. The views expressed in this article are those of the authors and are not necessarily those of the FSA, MRC, NIHR, or the UK Departments of Health.
